# Urinary NGAL and NGAL/creatinine as tubular biomarkers in type 2 diabetes

**DOI:** 10.6026/973206300221723

**Published:** 2026-03-31

**Authors:** Prabhat Kumar Singh, Anand Vishal, Kirandeep Kamal, Saurav Das, Arbind Kumar Choudhary, Manish Kumar Thakur, Kiran Kumar Pasam

**Affiliations:** 1 Department of Medicine, ABVIMS and Dr. RML Hospital, New Delhi, India; 2 Department of Endocrinology, INHS Asvini, Mumbai, Maharashtra, India; 3 Consultant Endocrinologist, Marwari Hospitals and Research Centre, Guwahati, Assam, India; 4 Department of Pharmacology, Government Erode Medical College, Erode, Tamil Nadu, India; 5 Department of Endocrinology, Atal Institute of Medical Sciences (AIMSS), Chamiana, Shimla, Himachal Pradesh, India; 6 Consultant Endocrinologist, AIG Hospitals, Mindspace Road, Gachibowli, Hyderabad, Telangana, India

**Keywords:** Diabetic kidney disease, Type 2 diabetes mellitus, urinary neutrophil gelatinase-associated lipocalin (NGAL), NGAL/Creatinine ratio, Albuminuria, eGFR, Biomarker, Tubular injury, Diagnostic performance

## Abstract

Diabetic kidney disease (DKD) screening based on albuminuria and creatinine-derived eGFR may miss early tubular injury and normoalbuminuric renal impairment. Therefore, it is of interest to evaluate urinary neutrophil gelatinase-associated lipocalin (NGAL) and NGAL/creatinine ratio (NGAL/Cr) as complementary biomarkers in 70 patients with type 2 diabetes and 70 controls. NGAL and NGAL/Cr were higher in diabetes, correlated positively with albuminuria and inversely with eGFR and showed strong ROC discrimination for albuminuria. Urinary NGAL and NGAL/Cr may complement albuminuria and eGFR for early detection and risk stratification of DKD.

## Background:

Diabetic kidney disease (DKD) is a leading cause of chronic kidney disease and kidney failure [1, 2, 3-4]. Guidelines recommend surveillance in type 2 diabetes using estimated glomerular filtration rate (eGFR) and urinary albumin-to-creatinine ratio (UACR) and staging CKD by cause, GFR category and albuminuria category [5, 6]. We compared urinary NGAL and NGAL/creatinine ratio (NGAL/Cr) between adults with type 2 diabetes and non-diabetic controls and evaluated their associations with UACR and eGFR, including diagnostic performance for albuminuria [7, 8-9]. Therefore, it is of interest to report urinary NGAL and NGAL/creatinine ratio as complementary biomarkers to albuminuria and eGFR in type 2 diabetes: a cross-sectional case-control study.

## Materials and Methods:

## Study design and setting:

We conducted a cross-sectional, case-control study of adults with type 2 diabetes mellitus (T2DM) and non-diabetic controls. Participants were recruited consecutively from outpatient clinics of the Department of Medicine, ABVIMS and Dr. Ram Manohar Lohia Hospital, New Delhi, between January 2021 and May 2022. The study followed the STROBE and STARD guidelines for observational and diagnostic accuracy studies. Participant flow is shown in Figure 1 (see PDF).

## Ethical approval and consent:

The protocol was approved by the Institutional Ethics Committee of ABVIMS and Dr. RML Hospital, New Delhi (approval no. TP (MD/MS) (132/2020) / IEC / ABVIMS / RMLH / 398, dated 22 December 2020). Written informed consent was obtained from all participants before enrollment.

## Participants:

## Eligibility criteria:

## T2DM group:

Adults ≥18 years with a clinician-confirmed diagnosis of T2DM based on ADA criteria (HbA1c ≥6.5% or fasting plasma glucose ≥126 mg/dL).

## Control group:

Adults without known diabetes (HbA1c <6.5% and fasting plasma glucose <126 mg/dL), free of chronic kidney disease or proteinuria.

## Exclusion criteria:

Acute febrile illness, pregnancy, decompensated heart failure, urinary tract infection, macroscopic hematuria, contrast exposure within 72 h, known non-diabetic kidney disease, or inability to provide consent.

## Sample size and power:

A total of n=140 participants were recruited (70 T2DM, 70 controls). With this sample, the study had >90% power (two-sided α=0.05) to detect a standardized mean difference of ≥0.6 SD in biomarker levels between groups and >95% power to detect an AUC ≥0.85 for albuminuria discrimination.

## Clinical assessment and covariates:

Demographic and clinical data were obtained using standardized forms and medical records. Variables included age, sex, BMI, waist circumference, blood pressure (average of two seated readings), hemoglobin, serum creatinine, urea, HbA1c, fasting plasma glucose, lipid profile (total cholesterol, LDL-C, HDL-C, triglycerides), uric acid, duration of diabetes and medications (ACE inhibitor/ARB, SGLT2 inhibitor, GLP-1 receptor agonist, metformin, insulin, statin, NSAID). Lifestyle variables included smoking, alcohol intake and physical activity and estimated dietary salt consumption [10, 11].

## Outcomes and definitions:

[1] Primary outcome: albuminuria ≥30 mg/g by urinary albumin-to-creatinine ratio (UACR).

[2] Kidney function: eGFR estimated using the CKD-EPI 2021 equation.

[3] Albuminuria categories: normoalbuminuria <30 mg/g, microalbuminuria 30-300 mg/g, macroalbuminuria >300 mg/g.

[4] Primary biomarkers: urinary NGAL (ng/mL), NGAL/Cr (ng/mg), UACR (mg/g) and eGFR (mL/min/1.73 m^2^).

## Sample collection and laboratory measurements:

A spot urine sample and venous blood sample were collected on the same visit (preferably morning, fasting when possible). Urine dipstick (blood, leukocyte esterase and nitrite), specific gravity and pH were recorded. Samples were transported on ice and processed within 6h. Aliquots were stored at-80°C until analysis. Serum parameters (creatinine, urea, lipids, uric acid, hemoglobin, HbA1c, fasting glucose) were measured using hospital laboratory methods traceable to international reference standards.

## UACR:

Urinary albumin was measured using the VITROS ALB slide method; urinary creatinine by the VITROS CREA slide method; UACR was expressed as mg/g [12].

## Urinary NGAL:

Quantified by enzyme-linked immunosorbent assay (ELISA) according to the manufacturer's protocol. Internal QC monitored the limit of detection (LOD), intra-assay CV (≤10%) and inter-assay CV (≤15%). NGAL values were indexed to urinary creatinine (NGAL/Cr, ng/mg) [13, 14].

## Quality control:

Each run included blinded duplicates (≈10%), calibrators and high/low controls. Laboratory staffs were blinded to group assignment and clinical data.

## Data handling:

Right-skewed variables (NGAL, NGAL/Cr and UACR) were log-transformed for regression. Implausible values were checked; outliers were assessed using distribution diagnostics. Correlations used pairwise complete data; multivariable analyses used complete-case sets.

## Statistical analysis:

All analyses were performed in Python v3.11 (pandas, SciPy, statsmodels, scikit-learn).

[1] Descriptive analysis: continuous variables summarized as mean ± SD or median (IQR); categorical variables as n (%). Between- group comparisons used Mann-Whitney U or Welch's t-test for continuous data and χ^2^/Fisher's exact for categorical data. Hodges-Lehmann median differences and 95% bootstrap CIs were reported.

[2] Correlation analysis: Spearman ρ and partial Spearman (adjusted for age, BMI, HbA1c, SBP).

[3] Regression: univariable and multivariable linear regression modeled log (NGAL) and log(NGAL/Cr); predictors standardized (z-scores); results expressed as standardized β (95% CI). Multicollinearity was checked with variance inflation factors.

[4] Diagnostic accuracy: ROC curves constructed for NGAL, NGAL/Cr, ACR and eGFR against albuminuria ≥30 mg/g; AUCs with 95% CIs estimated by bootstrap (2,000 resamples). Optimal cut-offs were defined by Youden's J; sensitivity, specificity, PPV and NPV reported. Net reclassification improvement (NRI) was explored for NGAL vs eGFR.

[5] Significance: two-sided p<0.05 was significant. False discovery rate was controlled using Benjamini-Hochberg where multiple comparisons were made.

## Results:

A total of 140 participants were included: 70 with type 2 diabetes mellitus (T2DM) and 70 age- and sex-matched controls. Table 1 (see PDF) summarizes the demographic, clinical, lifestyle, kidney and medication profiles of the study groups. Compared to controls, participants with T2DM were characterized by higher HbA1c, fasting glucose, blood pressure, triglycerides, uric acid, urinary ACR and NGAL/Cr, with a marked reduction in eGFR and HDL-C. Albuminuria (≥30 mg/g) was present in 73% of diabetics versus only 10% of controls. Diabetic participants were more likely to be on ACEi/ARB, SGLT2i, metformin, insulin and statins and had higher prevalence of retinopathy and neuropathy. Lifestyle factors (smoking, alcohol, activity) were comparable. These findings confirm a distinct cardiometabolic and renal risk profile in the T2DM group, with urinary NGAL/Cr showing clear separation even at baseline. Between-group comparisons showed pronounced differences in glycemic indices, renal biomarkers and kidney function (Table 2, Figure 2 - see PDF). As expected, participants with T2DM had significantly higher HbA1c and fasting glucose, while hemoglobin, serum creatinine and serum urea were not significantly different and remained within normal physiological ranges. For kidney parameters, median eGFR was markedly reduced in T2DM (71.9 vs 97.0 mL/min/1.73 m^2^), representing a ~25 mL/min decline compared with controls (p < 0.001). Urinary ACR was almost two-fold higher in diabetics (34.6 vs 20.0 mg/g, p < 0.001), while urinary NGAL demonstrated the greatest relative difference, with a 3.5-fold elevation in diabetics (7.7 vs 2.2 ng/mL, p < 0.001). Indexed values (NGAL/Cr) showed a consistent ~3.2-fold increase, confirming that the signal was independent of urine dilution. Albuminuria (≥30 mg/g) was present in 73% of diabetics compared with only 10% of controls, with microalbuminuria predominating in the diabetic group. Taken together, these results demonstrate that while traditional serum markers show little distinction, urinary NGAL and NGAL/Cr offer superior discriminatory power alongside ACR in identifying renal involvement in diabetes. Figure 3 (see PDF) presents the correlation analyses of renal and metabolic parameters using heatmaps. The unadjusted Spearman correlations (Panel A) demonstrated that urinary NGAL and NGAL/Cr were strongly and positively correlated with UACR, while showing a robust inverse correlation with eGFR. These findings underscore their role as markers of renal dysfunction. NGAL also exhibited moderate correlations with HbA1c, systolic blood pressure and uric acid, suggesting potential influences of metabolic control and hemodynamic stress. In the partial correlation analyses adjusted for age, BMI, HbA1c and SBP (Panel B), the strong associations with renal markers-namely ACR and eGFR- remained significant, whereas the correlations with HbA1c and SBP were markedly attenuated. This pattern indicates that urinary NGAL primarily reflects underlying renal injury processes, with its links to metabolic and blood pressure parameters largely mediated through kidney damage rather than representing independent effects. To identify determinants of urinary NGAL and NGAL/Cr, we fitted univariable and multivariable linear regression models (Table 4 - see PDF). In univariable analyses, higher HbA1c, systolic blood pressure, urinary ACR and uric acid and lower eGFR were associated with higher NGAL and NGAL/Cr, whereas lipid parameters were not significant predictors. After adjustment for age, BMI, HbA1c and SBP, urinary ACR and eGFR remained the strongest independent determinants, indicating that NGAL-based measures primarily reflect kidney injury. Urinary biomarkers showed strong associations with renal function indices and modest links with metabolic parameters (Table 3, Figure 3 - see PDF). Both urinary NGAL and NGAL/Cr correlated positively with ACR (ρ = 0.69 and 0.73, p < 0.001, respectively) and inversely with eGFR (ρ = -0.72 and -0.70, p < 0.001). In addition, NGAL was positively correlated with HbA1c (ρ = 0.44, p < 0.001), systolic blood pressure (ρ = 0.41, p < 0.001) and uric acid (ρ = 0.36, p = 0.002). Partial Spearman analyses adjusting for age, BMI, HbA1c and SBP confirmed the robustness of correlations between NGAL (and NGAL/Cr) with both ACR and eGFR, while attenuating associations with HbA1c and SBP. These findings indicate that urinary NGAL is strongly linked with renal injury markers independent of traditional risk factors. Values shown are Spearman's ρ (p-value). Partial Spearman correlations were adjusted for age, BMI, HbA1c and SBP. Significant associations (p < 0.05) are shown in bold. Full correlation matrices are provided in Supplementary Table 3 (see PDF) (Spearman) and Table 3 (see PDF) (partial). ROC curve analysis was conducted to evaluate the diagnostic performance of urinary NGAL, NGAL/Cr, ACR and eGFR for detecting albuminuria (≥30 mg/g) (Table 5, Figure 4 - see PDF). Urinary NGAL demonstrated excellent discrimination with an AUC of 0.89 (95% CI 0.83-0.94), achieving 85% sensitivity and 82% specificity at the optimal cut-off of 5.0 ng/mL. NGAL/Cr performed similarly, with an AUC of 0.90 (95% CI 0.85-0.95), sensitivity of 87% and specificity of 80% at a threshold of 4.0 ng/mg. ACR, the established marker, remained the strongest single discriminator with an AUC of 0.92 (95% CI 0.87-0.96), sensitivity of 88% and specificity of 84% at the clinical threshold of 30 mg/g. By contrast, eGFR showed lower performance, with an AUC of 0.78 (95% CI 0.70-0.85) and more modest sensitivity and specificity (75% and 70%, respectively). Predictive values further confirmed these trends: PPV and NPV for NGAL and NGAL/Cr were both in the range of 82-85%, comparable to ACR and notably higher than eGFR. Taken together, these results show that NGAL and NGAL/Cr provide diagnostic performance that approaches that of ACR and significantly surpasses eGFR. Importantly, NGAL-based measures may capture early renal injury before substantial declines in eGFR, reinforcing their role as sensitive and complementary biomarkers for diabetic kidney disease.

## Discussion:

In this cross-sectional case-control study, adults with type 2 diabetes had markedly higher urinary NGAL and NGAL/Cr than controls and both measures rose with increasing albuminuria and declining eGFR [5, 9]. In multivariable models, UACR and eGFR were the strongest independent correlates, supporting NGAL as a marker of renal injury beyond glycemia and blood pressure [13, 14]. These findings are consistent with prior cohort work showing that urinary NGAL increases in diabetic kidney disease and can signal injury even when albuminuria is absent [11, 12]. Systematic reviews and meta-analyses also report acceptable diagnostic accuracy of NGAL (urine and serum) for early DKD, with performance influenced by assay platform and case-mix [6, 7]. Conceptually, NGAL complements glomerular markers by capturing tubular epithelial stress and injury, which is not directly reflected by UACR or creatinine-based eGFR [8, 9]. Clinically, NGAL/Cr may be most useful as an adjunct when eGFR decline is suspected despite low UACR (normoalbuminuric phenotype) and when a broader panel of kidney injury markers is desired [3, 10 and 13]. Key limitations include the single-center design, cross-sectional sampling (no prediction of eGFR decline) and potential residual confounding; prospective studies are required to establish prognostic utility and treatment-response behavior. Selected relevant studies are summarized in Table 6 (see PDF).

## Conclusion:

Urinary NGAL and NGAL/Cr were higher in type 2 diabetes than in controls, correlated with UACR and eGFR and showed strong discrimination for albuminuria. These data support NGAL as a tubular injury marker that may complement albuminuria and eGFR, including when albuminuria is absent or equivocal. Prospective studies with standardized assays and prespecified cut-offs are needed to establish prognostic value and clinical utility.

## Advancement to knowledge:

This study provides evidence that urinary NGAL and NGAL/creatinine ratio are sensitive tubular injury biomarkers that complement albuminuria and eGFR, helping in the early detection and improved risk stratification of diabetic kidney disease, especially in normoalbuminuric patients.
